# Proposal for Low-Cost Optical Sensor for Measuring Flow Velocities in Aquatic Environments

**DOI:** 10.3390/s24216868

**Published:** 2024-10-26

**Authors:** Vinie Lee Silva Alvarado, Arman Heydari, Lorena Parra, Jaime Lloret, Jesus Tomas

**Affiliations:** 1 Instituto de Investigación para la Gestión Integrada de Zonas Costeras, Universitat Politècnica de València, Gandía C/Paranimf, 1, 46730 Grao de Gandia, Spain; vlsilalv@epsg.upv.es (V.L.S.A.); aheydar@doctor.upv.es (A.H.); jlloret@dcom.upv.es (J.L.); jtomas@upv.es (J.T.); 2 Departamento de Producción Agraria, Escuela Técnica Superior de Ingeniería Agronómica, Alimentaria y de Biosistemas, Universidad Politécnica de Madrid, 28040 Madrid, Spain

**Keywords:** LED-based sensor, aquatic sensors, prototype, turbidity, monitoring, marine ecosystems

## Abstract

The ocean, with its intricate processes, plays a pivotal role in shaping marine life, habitats, and the Earth’s climate. This study addresses issues such as beach erosion, the survival of propagules from species like *Posidonia oceanica*, and nutrient distribution. To tackle these challenges, we propose an innovative sensor that quantifies hydrodynamic velocity by measuring the output voltage derived from detecting changes in light absorption and scattering using LEDs and LDRs. Our results not only demonstrate the effectiveness of the sensor but also the accuracy of the processing algorithm. Notably, the blue LED exhibited the lowest mean relative error of 7.59% in freshwater, while the yellow LED was most precise in chlorophyll-containing water, with a mean relative error of 6.80%. In a runoff simulation, we observed similar velocities with the blue, green, and white LEDs, 6.89 cm/s, 6.99 cm/s, and 7.05 cm/s, respectively, for nearly identical time intervals. It is important to highlight that our proposed sensor is not only effective but also highly cost-efficient, representing less than 0.43% of the cost of a Nortek Vector 6 MHz and 0.18% of the Teledyne Workhorse II 300 kHz Marine. This makes it a key tool for managing marine ecosystems sustainably.

## 1. Introduction

Considering the inaccessibility and vastness of the areas that need to be monitored, overseeing human activities in the ocean has been a considerable challenge. These oceans, which comprise more than 70% of the Earth’s surface, are crucial for the lives of billions of people, as they provide food and facilitate global merchandise transport. Additionally, marine infrastructure related to wind energy, oil, and gas is vital for global energy supply [1,2].

The ocean exhibits processes that generally can only be resolved incompletely or approximately. Others, however, have been historically well-studied and have an analytical solution; for some more complex problems, approximation methods or numerical methods are used, which form the basis of computational models employed in research [3]. One of the critical parameters to be studied is velocity.

Flow velocity is of utmost importance. Among the most notable processes are those carried out by currents, which are responsible for the mixing of waters and the transport of heat, nutrients, sediments, pollutants, and planktonic organisms; this has a direct impact on marine organisms and their habitat, as well as on the Earth’s climate [4]. As a result, it is observed that the intensity of currents significantly influences the structure of ecosystems, as changes due to natural or anthropogenic factors directly affect water quality [5] and its biological richness.

Water velocity also affects the loss of seagrasses such as *Posidonia oceanica* [6] and the regression of beaches [7]. Velocity also impacts the development of other species, such as coho salmon (*Oncorhynchus kisutch*) and rainbow trout (*Oncorhynchus mykiss irideus*) [8].

Studying water flow in an estuary is critical due to the complex interaction between river currents and marine tides. Accurate monitoring of these flows using sensors can provide valuable data for understanding how these processes impact local biodiversity [9]. Current sensors for monitoring velocity are costly, which results in a low density of measurement points in the environment.

Therefore, there is a need for innovative and cost-effective tools and methods that allow for the accurate measurement of marine current velocities. These devices must also be accessible for research purposes, as precise data play a fundamental role in sustainable management and informed decision-making in marine and coastal ecosystems.

This work proposes the development of a low-cost sensor that measures hydrodynamic velocity in situ by detecting changes in light absorption and scattering. Light scattering is a physical principle in which wavelengths separate as they pass through a medium; it is utilized in the turbidity measurement methodology [10]. Light absorption occurs when a material retains certain wavelengths. Recently, this principle has been used to develop LED-based optical sensors rather than relying solely on scattered light [11]. The practical application of this sensor is significant, as it can be used for large-scale monitoring of water flow in marine and coastal ecosystems. Similar sensor systems exist for detecting fluid velocity based on temperature changes in the water caused by a resistor. The proposed sensor uses this principle but applies it to the optical characteristics of water, taking advantage of the considerable spatial and temporal variability in the optical properties of water in natural environments. It is proposed that optical sensors capable of tracking water turbidity every few milliseconds be used to obtain a variable turbidity signal. Another sensor with the same characteristics will be placed just a few centimeters away. Different light emitters and receivers will be used to detect turbidity. Our proposal is based on repeating these temporal changes in turbidity at the second sensor so that the time it takes for the same signal to be repeated at the second sensor is proportional to the water velocity. Therefore, the main objective of this work is to design a low-cost sensor capable of accurately quantifying water flow intended for large-scale monitoring.

The rest of the document is structured as follows: Section 2 reviews related methodologies and sensors. The development of the proposal and the sensor configuration are described in Section 3. Section 4 presents and analyzes the obtained results. Section 5 concludes the work, comparing the sensor with commercial alternatives and proposing improvements and future research.

## 2. State of the Art

Various tools and concepts were used to build a robust theoretical foundation in developing this work. This work is significant as it identifies and addresses deficiencies in monitoring flow velocity at sea, specifically through tracking changes in light absorption and scattering in the cross-sectional profile. A comprehensive review of the state of the art in various current meters was conducted, providing a complete view of current advancements and the challenges we face with the methodological implementation in optics using LEDs (Light Emitting Diodes) and LDRs (Light Dependent Resistors). This methodology is based on principles of Particle Image Velocimetry (PIV), an optical method for measuring the velocity distribution in fluids [12,13], and we utilize a float method for velocity calculation. This technology is non-invasive, as there is no direct interaction between the medium and the sensor [14]. Nevertheless, despite their accuracy and detailed flow visualization capabilities, traditional PIV systems tend to be costly and complex to deploy in natural aquatic settings.

This section presents various techniques and methodologies currently employed to study flow velocity in oceans and seas. Practical tools such as acoustic transducers, which transmit and receive acoustic signals to determine the speed and direction of marine flow, are highlighted. Similarly, the application of advanced electronics in methods like electromagnetic current meters is discussed. Both methods are relevant for continuous monitoring, emphasizing the practical and real-world applications in the sustainable management of marine ecosystems.

Zhao et al. [15] focus on designing a new underwater device for measuring ocean current velocities. The apparatus comprises a measurement boat, a cable, a fixed anchor, and an ocean current velocity detector. The detector employs a time-difference acoustic current meter, which is stable and widely used. This device allows for adjusting the detector’s depth according to actual requirements, facilitating the measurement of flow velocity at various depths.

Sarangapani [16] introduces a new Acoustic Doppler Current Profiler (ADCP) that employs a multi-frequency phased array transducer. This ADCP facilitates the simultaneous measurement of high-resolution current profiles in both the upper ocean or shallow waters and in the deep ocean up to 1500 m. Velocity resolution measurements are enhanced by correlating measurements at 38 and 300 kHz. Results are presented regarding range and velocity measurements for the phased array transducers at two different frequencies.

Gytre et al. [17] employ three types of ultrasound current meters: one Doppler current meter and two time-of-flight current meters, each handling different sampling volumes. The focus is on the significance of turbulence in the predator-prey interactions of plankton, as it increases the contact rate between these relatively immobile organisms. Direct turbulence measurements were conducted in Lofoten, Norway, where turbulent mixing is primarily generated by tidal and wave-induced mixing. The results indicate that both instruments can be used to resolve small-scale turbulence. However, Doppler noise limits the performance of the Doppler current meter, while the length of the acoustic path restricts the performance of the time-of-flight current meters.

MacVicar et al. [18] conducted two field tests to compare the performance of an Electromagnetic Current Meter (ECM) and an Acoustic Doppler Velocity Meter (ADV) in gravel-bed rivers. Measurements were taken at two sites with varying velocities and turbulence intensities. The results showed a general agreement between the ECM and ADV, although with some limitations. At high turbulence intensities, the ADV exhibited anomalous behavior. Errors in the ADV measurements were estimated using four different methods. The main conclusion is that the older ECM technology provides more reliable estimates of flow parameters in conditions of high turbulence.

Fu [19] quantifies the instantaneous measurement uncertainty and mean velocity with PIV, calculated by the average displacement of pixels. Similarly, Li et al. [20] conducted a 3D-PIV sweep applied to visualize transverse flows, obtaining a model that allows for the examination of flows in microscopic systems.

The development of sensors capable of measuring this parameter has been the subject of numerous studies presented by academic and research institutions worldwide. Furthermore, efforts have been made to apply new technologies to improve accuracy or to pursue more cost-effective alternatives [21,22,23,24]. As no prior research or similar projects using LEDs and LDRs for pattern detection and water flow calculation have been identified, various evaluations are necessary to determine the sensor’s optimal configuration [25]. This involves conducting exhaustive tests to calibrate the components, validate the results, and compare them with conventional methodologies [26]. These activities are essential to ensure the accuracy and reliability of the data collected during the development of the proposed system. This contributes to a relevant and actively researched field of study.

## 3. Materials and Methods

This section explains the sensor’s design, development, assembly, and programming for measuring flow velocity.

### 3.1. Background

A flow sensor is being developed to measure flow velocity in coastal waters. Alongside traditional methods, an alternative approach involves using a turbidity sensor based on a system of LEDs and LDRs. This low-cost system detects changes in the intensity of light reflected by particles in the water, which can then be processed. By recording these values across one section and looking for similar patterns in another section at a known distance, the time can be calculated, allowing the flow to be determined. Using neuromorphic networks was considered for estimating flow velocity and analyzing water composition [27]. However, due to the high processing demands and specialized hardware required, a simpler and more feasible approach was prioritized in the initial stages. The technology for measuring flow, which relies on identifying variations in light absorption and scattering, is considered an affordable and straightforward infrastructure solution that facilitates accurate data collection. Because the equipment needed for these measurements needs to be installed steadily, this methodology must be employed in the vicinity of the benthic zone. The seabed is found at the bottom of the ocean, or the benthic zone. Because of its stability, this area is perfect for installing equipment and conducting ongoing monitoring. Strict control over the variables involved in the process dramatically aids in detecting and rectifying systematic or random errors. Among these variables are as follows:

### 3.2. Testing

The system’s minimum flow rate must be considered; to determine this, it is calculated using Equation (1), as it will be measured by flow rate.

(1) Q=u×A

where *Q* is the flow rate, *u* is the average fluid velocity over the time intervals during which the flow measurement is initiated using the volumetric method, and *A* is the cross-sectional area with a constant value of 5.07 cm^2^.

### 3.3. System Velocity

In order to calculate the system’s velocity, various flow meters were considered, such as the rotameter; however, it is mainly suited for vertical flows and has limitations in horizontal flows, which are common in marine environments. Alternatives like turbine meters are ideal but incur energy losses during fluid transport, while acoustic methods are expensive. Therefore, we chose the 1000 mL volumetric flask method, which effectively and economically measures low velocities with minimal measurement errors due to the large volume. The velocity equation was calculated based on the sampling time to ensure that the system’s velocity and conditions remained constant during sampling. This was performed by tabulating values over different time intervals, as shown in Table 1. Using the flow measurement method, the measurement time is the calculated time to fill the volume of the flow measurement container. Velocity is calculated based on the flow rate and the cross-sectional area from Equation (1). The time was determined by subtracting half the flow measurement time from the total time, as the velocity varies over time. Since the relationship between velocity and time is linear, this approach ensures an accurate and consistent calculation.

#### Error Propagation

For these measurements, various analog and digital devices were used, including a measuring cylinder (minimum resolution = 10 cm^3^), a caliper (minimum resolution = 0.01 cm), and a stopwatch (minimum resolution = 0.01 s). In this context, velocity is not measured directly but is derived from three distinct variables: the measured volume of water, the diameter of the pipe, and the flow measurement time. This formula involves products and quotients of these variables, each with its own measurement error. Therefore, error propagation is calculated to determine how individual uncertainties affect the total error in the system’s velocity. For this particular case, logarithmic error propagation is applied to estimate the absolute error of the velocity [28]. The velocity equation is defined in Equation (2), as follows:

(2) $$u=\frac{4\times V}{\pi \times D^{2}\times t}$$

where π is the constant pi, *D* is the diameter, *V* is the volume of displaced fluid, *t* is the elapsed time, and *u* is the flow velocity.

The natural logarithm is taken of each term in the equation, considering the properties of logarithms; see Equation (3).

(3) $$u=-\ln\frac{\pi }{4}\ln V-\ln D^{2}-\ln t$$

Differentiate concerning the corresponding variables and solve for *du*, yielding Equation (4):

(4) $$du=u\left(\frac{dV}{V}-2\frac{dD}{D}-\frac{dt}{t}\right)$$

Next, the differential elements are identified as errors, changing the signs of those that are negative, resulting in Equation (5).

(5) $$∆u=u\left(\frac{∆V}{V}+2\frac{∆D}{D}+\frac{∆t}{t}\right)$$

where Δ*u* is the absolute error in velocity, Δ*D* is the error in diameter, which is 0.01 cm due to the digital precision of the instrument used, Δ*V* is the volume error, with a value of 0.005 cm^3^ associated with the analog error of the device, and Δ*t* is the error in time, estimated at 0.01 s, also due to the digital precision of the measuring tool.

**Figure 1:**
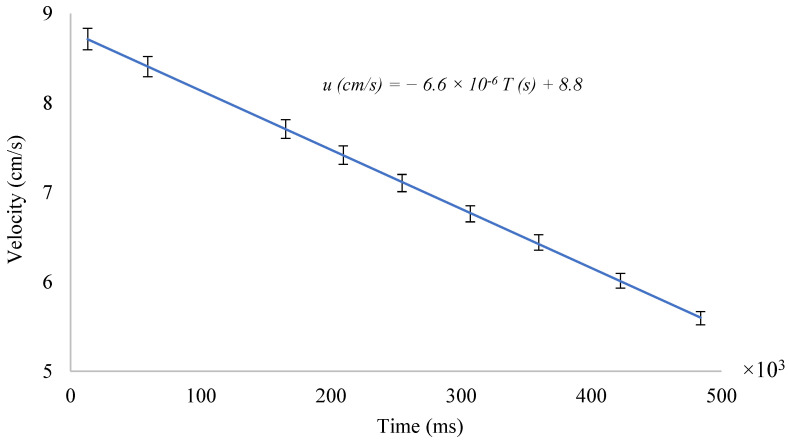
Behaviors of velocity in the system.

The value of the pipe’s internal diameter is constant across the cross-section (2.54 cm). The measured volume and measurement time will be taken from Table 1. Figure 1 shows the absolute error results in each measurement, displaying the general trend of velocity and the associated errors.

### 3.4. Origins of Turbidity

Table 2 includes the turbidity of the fluids used. The commercial turbidity meter model TU-2016 assessed the sensor’s maximum efficiency levels.

### 3.5. Tracer

An extract of *Lactuca sativa* was prepared with a concentration of 0.71 g of leaves per milliliter of water to simulate suspended solids in the ocean and chlorophyll. This fluid affects the LDR readings. The tracer was introduced into the system at 20 mL for three seconds, with a two-second interval between each introduction until sampling was complete.

### 3.6. Setup

**Figure 2:**
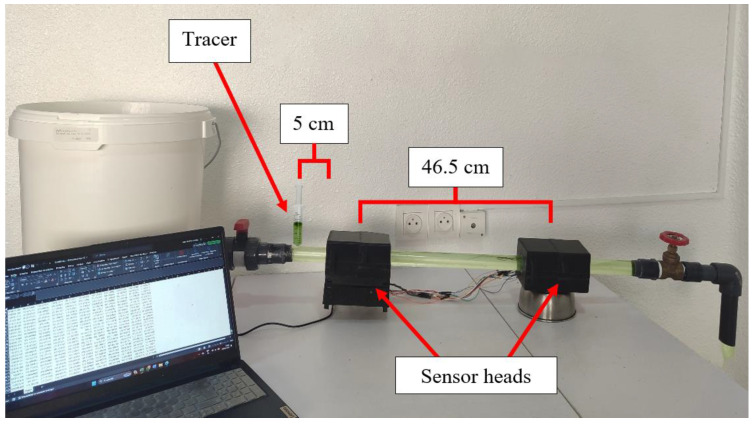
Readings in the proposed system are taken using water with chlorophyll.

Figure 2 illustrates the experimental sampling process and sensor operation. It shows the layout of the experimental system, with the tracer positioned 5 cm before the first sensor head. This sensor head is spaced 46.5 cm from the second sensor head, considering the central axes of both. The system operates with an Arduino Mega 2560 Rev3 [29], which performs the measurement and sends the data to the computer in real-time.

**Figure 3:**
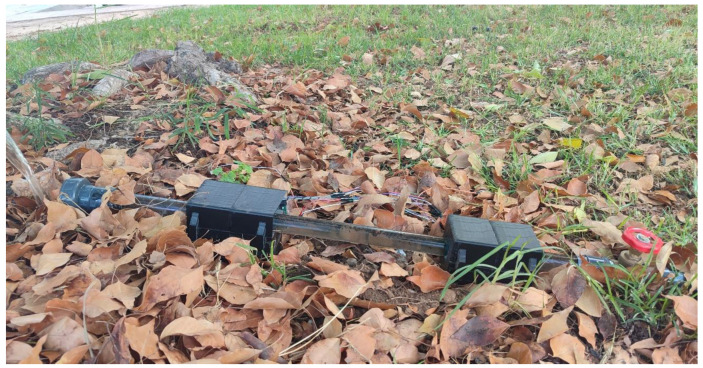
Runoff simulation system.

In addition to the measurements for calibrating the equipment and model, the efficiency is tested by simulating potential runoff to take readings and process them, as shown in Figure 3. The obtained readings were expressed in voltages in response to light absorption and scattering changes.

## 4. Proposal

A specific set of electronic components was used to develop a flow sensor that measures changes in light absorption and scattering in aquatic environments. The purpose and function of each of these elements in the context of the sensor design are explained below.

### 4.1. Sensor Components

#### 4.1.1. Arduino Mega 2560 Rev3

The Arduino Mega 2560 Rev3 is a microcontroller board based on the ATmega2560. This microcontroller is well-suited for this sensor, as it requires numerous digital and analog inputs and outputs to control and read multiple LEDs and LDRs. The board meets the requirements to handle the ten pairs of LEDs and LDRs.

#### 4.1.2. Light Emitters and Detectors

LEDs are used in the sensor as light sources. When turned on, they emit a beam of light that passes through the fluid in the pipe. The light may be absorbed or scattered by particles in the water, which will be detected by the corresponding LDRs, whose electrical resistance varies according to the amount of light falling on them. Using ten LEDs and LDRs allows for the creation of 5 measurement pairs. These pairs are strategically distributed along the pipe, meaning they are placed at specific intervals calculated to ensure variations in flow and particles at different points are captured. This strategic distribution helps reduce errors and ensures accurate fluid measurement.

The LEDs will relate to 220 Ω resistors to limit the current passing through them. Since LEDs are sensitive to current, these resistors protect them from potential damage by regulating the current to a safe level. On the other hand, the LDRs will be used in conjunction with 22 kΩ resistors to form a voltage divider. This divider allows changes in the LDR’s resistance, caused by variations in the light received, to be translated into a voltage change that the Arduino can read. This resistor maximizes the range of analog values [27], with a broader measurement range enabling more accurate and resolved readings.

#### 4.1.3. Enclosure

The enclosure forms part of the structural support and mounting for the LEDs and LDRs, keeping them in the strategic and precise positions required. Additionally, it prevents or reduces the influence of ambient light on the readings from the LDRs.

### 4.2. Sensor Design

**Figure 4:**
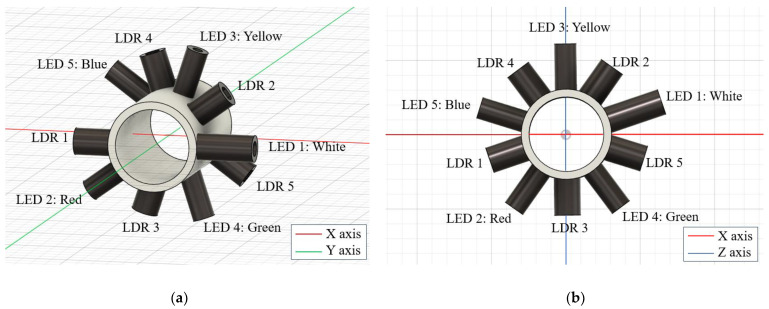
(**a**) Distribution of the LEDs and LDRs along the pipe. (**b**) Front view, rotated configuration.

The sensor’s design for the precise measurement of water flow velocity is based on the strategic arrangement of pairs of LEDs and LDRs along the cross-section of a pipe. Each pair is separated by 1 cm on the Y-axis, measured from the outer surface of the housing to the pipe (depending on the size of the LED), and is arranged with an inclination of 36 degrees, allowing for thorough and sensitive monitoring of the optical properties of the flow, as graphically depicted in Figure 4.

#### Initial Configuration

The first pair of LED and LDR is initially positioned in the horizontal plane of the pipe. Here, the LED is placed at 0 degrees on the X-axis, while the LDR is positioned precisely opposite at 180 degrees in the same horizontal plane. This alignment ensures that the LDR accurately detects the light emitted by the LED through the water flow. Each pair of sensors is moved along the Y-axis to capture data at different sections and angles of inclination:

- Second Pair: Located 1 cm deep on the Y-axis, the LDR is inclined at 36 degrees relative to the horizontal axis, while the LED is at 216 degrees.
- Third Pair: Positioned 2 cm deep on the Y-axis, the LED is oriented at 72 degrees and the LDR at 252 degrees.
- Fourth Pair: Placed 3 cm deep on the Y-axis, the LDR is 108 degrees, and the LED is at 288 degrees.
- Fifth Pair: The final pair, located 4 cm deep on the Y-axis, has the LED positioned at 144 degrees and the LDR at 324 degrees.
- Finally, a rotation of 18 degrees along the Y-axis is made to divide the distribution of LEDs and LDRs conveniently. The rotated configuration is shown in Figure 4b.

### 4.3. Technical Advantages

Each pair of LEDs and LDRs operates in opposition to minimize interference between consecutive measurements, ensuring the accuracy of light absorption and scattering readings. The sensor configuration facilitates installation, maintenance, and data collection in underwater environments, ensuring reliability and durability in industrial and environmental applications. Additionally, it offers several advantages.

#### 4.3.1. Comprehensive Cross-Sectional Coverage

The distribution of the sensors in five pairs, each separated by 36 degrees and positioned at different levels (Y-axis), ensures comprehensive coverage of the pipe’s cross-section. This arrangement guarantees that changes in light absorption and scattering are detected at multiple points, providing a detailed and accurate profile of the conditions within the pipe. This is crucial for obtaining precise data on water flow.

#### 4.3.2. Minimization of Interference Between Sensors

By positioning the LEDs and LDRs in opposing pairs, staggered vertically by 1 cm, interference between adjacent sensors is minimized. This arrangement ensures that the measurement from one pair does not affect the accuracy of the readings from other pairs, thereby improving the reliability and precision of the collected data.

#### 4.3.3. Adaptability of the Velocity Range to the Proposed System

Reducing the distance between each pair of sensors to 1 cm along the Y-axis strategically decreases the sensor’s size while maintaining an appropriate range of flow velocities for the system. This optimized spacing not only minimizes light interference between adjacent LEDs but also enhances the detection of subtle changes in light absorption and scattering, thereby improving the sensor’s precision in monitoring water flows at varying speeds. Data recorded every 10 ms and a 1 cm separation between LEDs ensure that each LED–LDR pair provides between 9 and 10 readings before the flow reaches the next pair, establishing a maximum operational speed of 11 cm/s.

#### 4.3.4. Optimization of Space Within the Pipe

The 36-degree inclination between each sensor pair allows for a compact and efficient arrangement, making the most of the available space. This is particularly advantageous in applications where space is limited and a setup that does not interfere with the natural water flow is required.

### 4.4. Sensor Circuit

**Figure 5:**
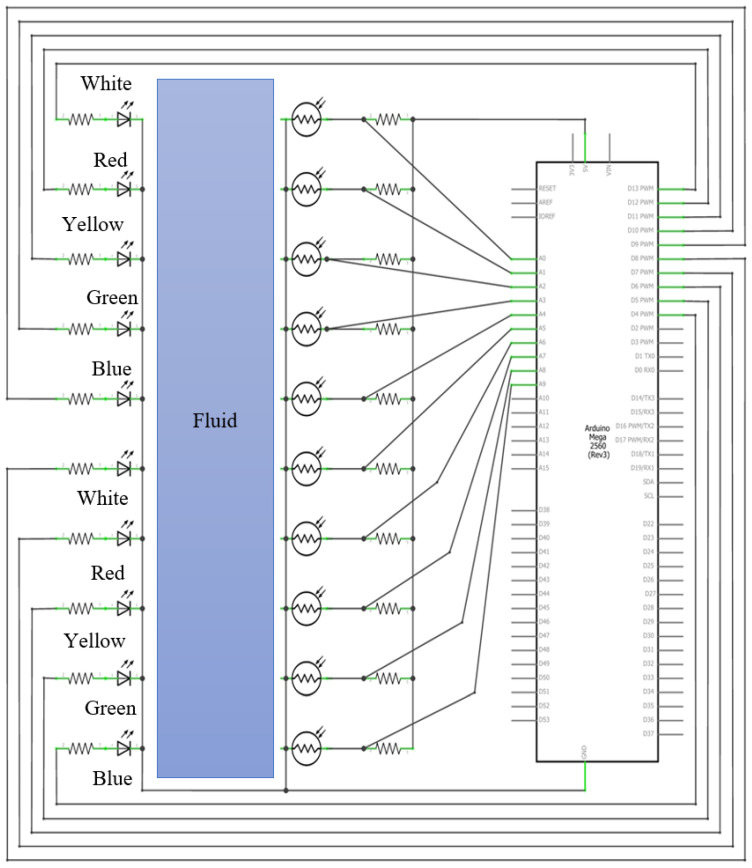
The electrical circuit of the proposed sensor.

The programming of this sensor is carried out on an Arduino Mega 2560 Rev3 due to the need for multiple inputs for the LEDs and LDRs based on the electrical circuit shown in Figure 5.

The sensor is based on a set of LEDs and photodiodes (LDRs) to measure the flow velocity in a pipe. The LEDs emit light at different wavelengths (white, red, yellow, green, and blue), and the LDRs detect the amount of light scattered or absorbed by the fluid flowing through the pipe. The process begins with activating the LEDs, which reach their maximum intensity. Once the LEDs are at maximum intensity and the LDRs have stabilized, the LDRs start taking measurements every 10 ms. These measurements are taken in two sections of the pipe (heads). Data are collected from each head’s five pairs of LEDs and LDRs. These data are compared to identify patterns in the changes in light absorption and scattering, which allows for the calculation of the time it takes for the tracer or suspended solids to pass from one section to the other. Once the patterns are detected, the detection times of the tenth LDR are subtracted from those of the fifth LDR. The flow velocity can be determined by the distance between the two heads and the processed time. This sensor leverages the properties of light and its interaction with fluids to measure flow velocity in a non-invasive manner. Its design and configuration are justified by its ability to provide fast and accurate measurements using relatively simple and affordable components.

### 4.5. Housing Design

**Figure 6:**
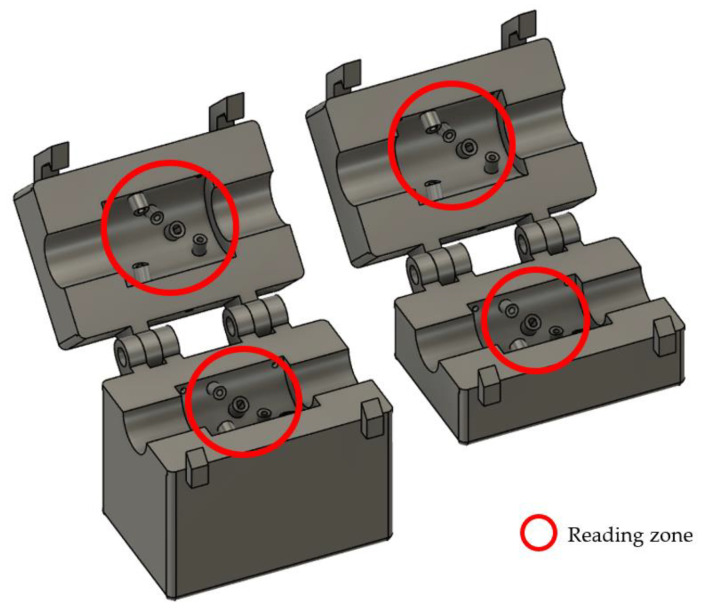
Three-dimensional structural design of the sensor housing.

Based on the technical considerations described in the previous section, Figure 6 shows the final design of the sensor housing, which is designed to house the electronic components, reduce external influence on the measurements, and protect the electronic circuit from water. A modular design with hinges is observed to facilitate opening and closing. This design ensures precise alignment of the heads for measurement, with each head containing five pairs of LEDs and LDRs covering the entire cross-section of the pipe. Additionally, each LED and LDR is strategically placed according to the distribution shown in Figure 4.

The parts were in Autodesk Fusion 360 [30]. Additionally, the modified sections were finalized in SolidWorks [31]. The parts were manufactured using a Sidewinder X2 3D printer. DLP filaments with a diameter of 1.75 mm and a 0.4 mm nozzle were used for the part construction. The build temperature for the parts was 200 °C and the bed temperature was 70 °C. The approximate build time for the parts was 70 h. Approximately 200 m of filament was used in the part manufacturing process.

### 4.6. Data Acquisition and Processing

The turbidity distribution is used as an indicator of the local fluid velocity; however, dispersion phenomena caused by turbulence and transport can lead to variations in tracer concentrations. Although light absorption and scattering often exhibit similar characteristics between the stations, the conditions at each can alter the output voltage, meaning there is not always a direct correspondence. To compare the signals from the two stations over different time intervals (the time it takes for a signal to pass to the next head), we considered time series analysis methods that allow for the evaluation of the correlation between the data. Nonetheless, this approach requires additional hardware resources for processing; therefore, an analysis was chosen that calculates the moments when the readings drop.

In order to obtain the data, an Arduino Mega 2560 Rev3 was used. Algorithm A1, see Appendix A, describes the data collection process, which began with the sequential activation of all LEDs, allowing each LDR to stabilize its readings and wait for cyclic instructions to read the analog values of each at regular time intervals, considering the code processing time (10 ms). These data were temporarily stored in the microcontroller’s memory and later exported to a CSV file for easier processing and analysis.

With Matlab R2024b [32], the readings were converted to voltage using Algorithm A2. Algorithm A3 employed the “detrend” command to remove the constant trend. This process involves subtracting the average from each value and then adjusting negative values to zero. These algorithms can be seen in Appendix A.

This procedure resulted in data matrices where some contained significant voltages while others contained zeros. Given the enormous volume of data, this approach also helps to disregard voltages from readings affected by tracer diffusion. With Algorithm A4 (see Appendix A), 3000 local maxima were identified in the “data” matrix, retaining all data corresponding to these maxima. The data were conveniently organized with time in the first column, followed by columns corresponding to a single color. Only the data with at least one reading in either of the two columns for the same row were retained to improve accuracy.

Moreover, the system operates with speeds ranging from 5 cm/s to 11 cm/s, so the data will be restricted to this range. Algorithm A5 is essential for the analysis, as it accurately detects the moment when the readings drop to zero, indicating that the tracer has completely passed. It calculates these specific time instants at both sensor heads, and, given the known distance between them, the velocity is determined.

## 5. Results

This section describes the calibration and data processing of the sensor, examining measurements taken in various types of water, including potable water and water with chlorophyll. This allows for evaluating the sensor’s accuracy under different environmental conditions, and it was also tested under semi-real conditions, simulating runoff.

### 5.1. Measurements in Potable Water

The fluid used in the first measurement was potable water. Since potable water has a turbidity of 0 NTU, the light absorption and scattering changes at different wavelengths (colors) are highly pronounced. This turbidity characteristic provides high-resolution readings, enabling precise evaluation of light fluctuations. It also simplifies developing and studying the algorithm needed to estimate the tracer’s velocity at each moment.

**Figure 7:**
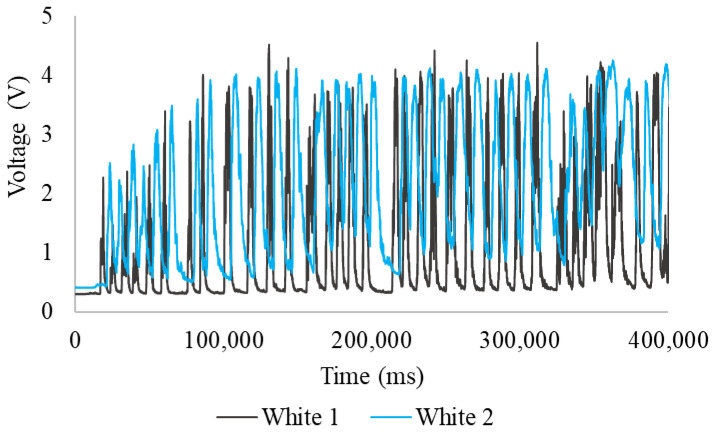
Readings during sampling periods, white LED, potable water.

Figure 7 is the result of applying Algorithms A1 and A2. It shows voltages ranging from a minimum of 0 V to a maximum of 5 V. These voltages result from changes in the absorption and scattering of light from the white LED during the sampling period (400,000 ms) caused by water flow. These readings were recorded as a function of time, measured in milliseconds (ms). The graph fluctuations represent specific moments when the tracer moves through the sensor’s reading zone. During these instances, changes in the absorption and scattering of light detected by the sensor are observed, leading to fluctuations that generate sharp curves.

Additionally, there is a noticeable similarity between the data series captured, though with a clear time offset between series 1 (first head) and series 2 (second head). This time offset is a critical factor for calculating the tracer’s velocity. In the readings from the first head (White 1), a maximum of 4.55 V, a minimum of 0.29 V, and an average of 1.09 V were recorded. Meanwhile, the second head (White 2) recorded a maximum of 4.25 V, a minimum of 0.41 V, and an average of 2.10 V.

#### Expanded Readings of Potable Water

**Figure 8:**
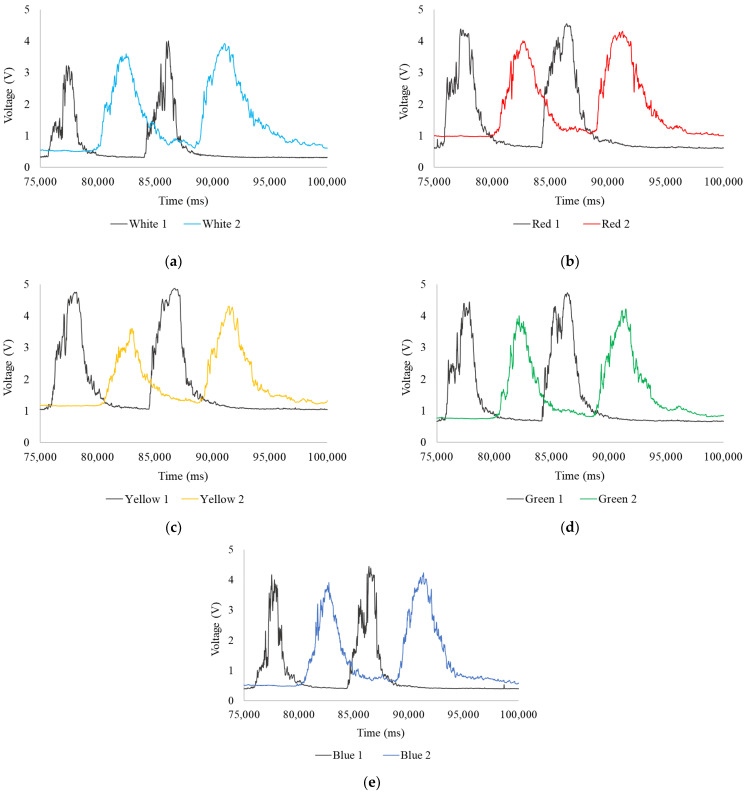
Expansion of readings from 75,000 ms to 100,000 ms, potable water: (**a**) white LED; (**b**) red LED; (**c**) yellow LED; (**d**) green LED; and (**e**) blue LED.

Figure 8 shows the voltages resulting from changes in light absorption and scattering between 75,000 ms and 100,000 ms. The measurements highlight the time offset between the curves with good resolution as fluctuations increase, generating two sharp curves. In Figure 8a, the first head’s (White 1) readings show a maximum of 3.22 V, a minimum of 0.32 V, and an average of 1.15 V for the first curve, and a maximum of 4.01 V, a minimum of 0.33 V, and an average of 1.06 V for the second; the second head (White 2) records a maximum of 3.60 V, a minimum of 0.63 V, and an average of 2.49 V for the first curve, and a maximum of 3.92 V, a minimum of 0.63 V, and an average of 1.90 V for the second. In the first head, the curve starts at minimum values, reaches several peaks, and then rapidly falls back to minimum values. In contrast, the second head’s curve shows a gradual decline and greater temporal dispersion. This curve broadening can be attributed to tracer diffusion along the pipe section due to turbulent flow and system accessories.

When the tracer is introduced into the fluid, it progressively disperses, causing its concentration to be non-uniform at any given moment, leading to fluctuations in the readings. The tracer affects each point of the sensor differently, prolonging the readings over time. The first head’s readings show a sharp, narrow drop because the tracer does not have enough time to diffuse, or the transport phenomena do not immediately affect it. In contrast, the second head gradually declines, as the tracer does not pass through the sensor instantly. This difference in curve decline directly reflects the tracer’s diffusion and visually indicates how the tracer concentration varies over time and across the pipe’s cross-section.

In Figure 8b, the first head (Red 1) shows a maximum of 4.38 V, a minimum of 0.61 V, and an average of 2.20 V for the first curve, and a maximum of 4.55 V, a minimum of 0.63 V, and an average of 1.98 V for the second; the second head (Red 2) records a maximum of 4.01 V, a minimum of 1.00 V, and an average of 2.60 V for the first curve, and a maximum of 4.31 V, a minimum of 1.06 V, and an average of 2.22 V for the second. In Figure 8c, the first head (Yellow 1) shows a maximum of 4.75 V, a minimum of 1.04 V, and an average of 2.67 V for the first curve, and a maximum of 4.87 V, a minimum of 1.06 V, and an average of 2.37 V for the second; the second head (Yellow 2) records a maximum of 3.61 V, a minimum of 1.17 V, and an average of 2.28 V for the first curve, and a maximum of 4.31 V, a minimum of 1.25 V, and an average of 2.23 V for the second. A noticeable decrease in the maximum readings from the second head explains the diffusion process of the tracer in the fluid. In Figure 8d, the first head (Green 1) shows a maximum of 4.44 V, a minimum of 0.66 V, and an average of 2.03 V for the first curve, and a maximum of 4.73 V, a minimum of 0.68 V, and an average of 1.95 V for the second; the second head (Green 2) records a maximum of 4.01 V, a minimum of 0.78 V, and an average of 2.43 V for the first curve, and a maximum of 4.22 V, a minimum of 0.82 V, and an average of 1.88 V for the second. Finally, in Figure 8e, the first head (Blue 1) shows a maximum of 4.17 V, a minimum of 0.40 V, and an average of 1.39 V for the first curve and a maximum of 4.45 V, a minimum of 0.41 V, and an average of 1.31 V for the second; the second head (Blue 2) records a maximum of 3.91 V, a minimum of 0.51 V, and an average of 2.08 V for the first curve, and a maximum of 4.24 V, a minimum of 0.63 V, and an average of 1.72 V for the second.

### 5.2. Measurements in Water with Chlorophyll

**Figure 9:**
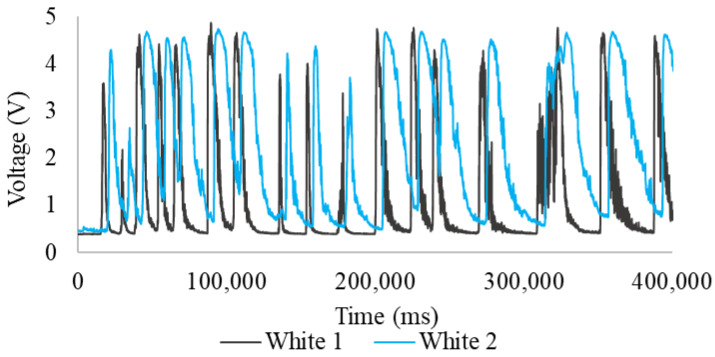
Readings during the sampling time, white LED, water with chlorophyll.

The fluid used in the second measurement was water with chlorophyll. This water has a turbidity of 10 NTU. Figure 9 is the result of applying Algorithms A1 and A2. It shows the voltages ranging from a minimum of 0 V to a maximum of 5 V. These voltages result from changes in the absorption and scattering of light from the white LED during the sampling period (400,000 ms). A notable similarity is observed between the captured data series, highlighting the time offset. However, it is important to note that in the second reading, the values again do not usually decrease immediately after the tracer has passed. This observation indicates that the tracer tends to mix and affect the resolution of readings. This is a crucial component to consider when improving the system, especially when reducing the likelihood of anomalies and ensuring greater measurement accuracy. These anomalies in the readings can not only make calculations more difficult but also produce results that differ from actual values, potentially compromising the model’s accuracy. The fluctuations generate 17 very sharp curves in both heads; in the first head (White 1), the maximum is 4.87 V, the minimum is 0.39 V, and the average is 1.34 V, while the second head (White 2) has a maximum of 4.74 V, a minimum of 0.44 V, and an average of 2.38 V.

#### Expanded Readings of Water with Chlorophyll

**Figure 10:**
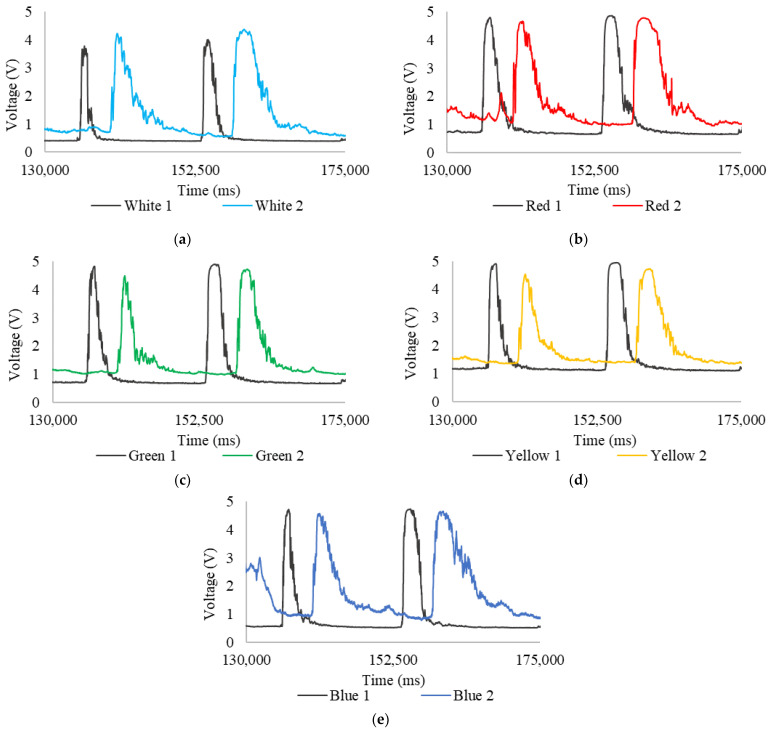
Expanded readings from 130,000 ms to 175,000 ms, water with chlorophyll: (**a**) white LED; (**b**) red LED; (**c**) yellow LED; (**d**) green LED; and (**e**) blue LED.

Figure 10 shows the voltages resulting from changes in light absorption and dispersion between 130,000 ms and 175,000 ms. The measurements allow for a clear resolution of the temporal offset between the curves with fluctuations increasing and generating two very pronounced curves. In Figure 10a, for the readings corresponding to the first head (White 1), the first curve has a maximum of 3.78 V, a minimum of 0.43 V, and an average of 1.83 V; while the second has a maximum of 4.01 V, a minimum of 0.40 V, and an average of 2.25 V; for the second head (White 2), the first curve has a maximum of 4.22 V, a minimum of 0.73 V, and an average of 2.07 V; while the second has a maximum of 4.38 V, a minimum of 0.59 V, and an average of 2.84 V. In Figure 10b, for the readings corresponding to the first head (Red 1), the first curve has a maximum of 4.80 V, a minimum of 0.75 V, and an average of 3.57 V; while the second has a maximum of 4.86 V, a minimum of 0.66 V, and an average of 3.74 V; for the second head (Red 2), the first curve has a maximum of 4.67 V, a minimum of 1.05 V, and an average of 2.56 V; while the second has a maximum of 4.79 V, a minimum of 1.02 V, and an average of 3.42 V. In Figure 10c, for the readings corresponding to the first head (Yellow 1), the first curve has a maximum of 4.91 V, a minimum of 1.19 V, and an average of 3.43 V; while the second has a maximum of 4.95 V, a minimum of 1.13 V, and an average of 3.84 V; for the second head (Yellow 2), the first curve has a maximum of 4.54 V, a minimum of 1.37 V, and an average of 2.45 V; while the second has a maximum of 4.73 V, a minimum of 1.40 V, and an average of 3.26 V. In Figure 10d, for the readings corresponding to the first head (Green 1), the first curve has a maximum of 4.84 V, a minimum of 0.75 V, and an average of 3.10 V, while the second has a maximum of 4.91 V, a minimum of 0.70 V, and an average of 3.61 V; for the second head (Green 2), the first curve has a maximum of 4.50 V, a minimum of 1.06 V, and an average of 1.99 V; while the second has a maximum of 4.74 V, a minimum of 1.03 V, and an average of 2.79 V; it is observed that, at this wavelength, the readings from the second head show less temporal diffusion in the maximum values of the curve. In Figure 10e, for the readings corresponding to the first head (Blue 1), the first curve has a maximum of 4.72 V, a minimum of 0.56 V, and an average of 2.72 V, while the second has a maximum of 4.74 V, a minimum of 0.53 V, and an average of 3.40 V; for the second head (Blue 2), the first curve has a maximum of 4.58 V, a minimum of 0.91 V, and an average of 2.39 V; while the second has a maximum of 4.66 V, a minimum of 0.87 V, and an average of 3.30 V; furthermore, it is evident that at this wavelength the readings fluctuate much more.

### 5.3. Calibration Results with Potable Water

Using Algorithms A3–A5 allowed for the reduction of large amounts of data and the calculation of predicted velocity. The true velocity is the one calculated with the regression line from Figure 1 and is shown in Equation (6). The results show a positive slope across all series, indicating that the proposed algorithm captures the general trend of the data. However, there is greater dispersion at velocities below 6 cm/s, suggesting that these data were obtained close to the lower limit at which the system could predict velocity, which is 5 cm/s. As a result, the predictions tend to be less accurate, underestimating at lower velocities and overestimating at higher velocities. It should be noted that there is a varying range of velocities among the colors due to the turbidity of the waters, which potentially makes them more sensitive to detection at certain wavelengths.

(6) $$u_{\mathrm{True}}\;(\mathrm{cm}/s)=-0.0000066\;\times \;\mathrm{Time}\;(s)+8.8$$

**Figure 11:**
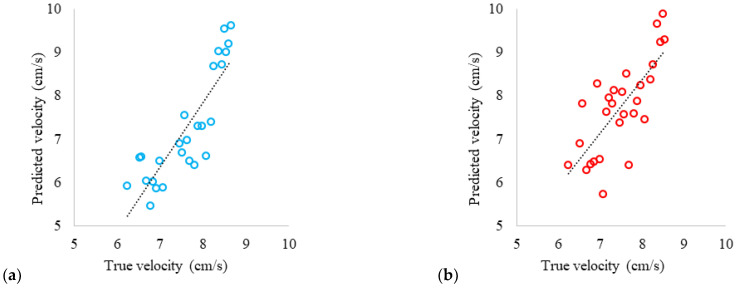

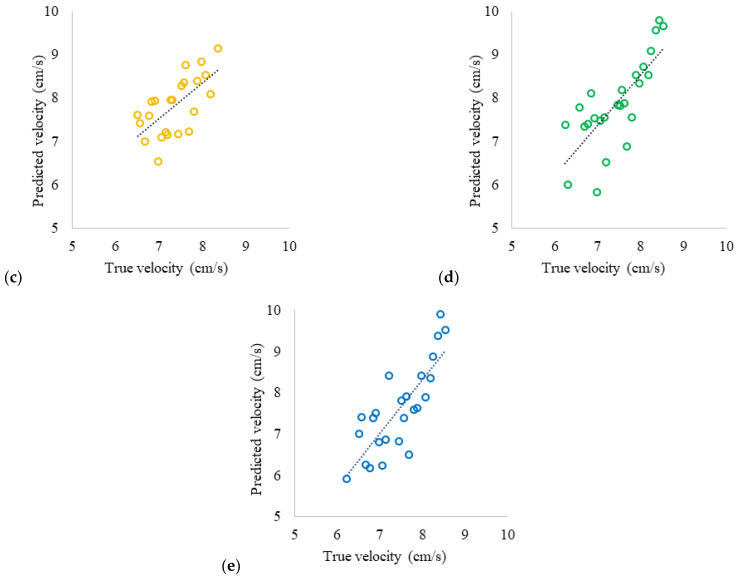
Predicted velocity vs. true velocity, white, potable water: (**a**) white LED; (**b**) red LED; (**c**) yellow LED; (**d**) green LED; and (**e**) blue LED.

In Figure 11, readings at different wavelengths are observed, with points clustering near the regression line. The accuracy of measurements made with the white LED (Figure 11a) is an important factor to consider; it shows less dispersion in the data, especially at higher velocity ranges; this indicates that the use of a white LED improves the accuracy of the system’s velocity detection and reduces deviations at high velocities; this may be due to the white light covering all wavelengths of the visible spectrum; the regression line has an R^2^ = 0.7368, which explains 73.68% of the variability in the data; overall, although the model performs well, accuracy could be improved by optimizing measurement conditions, especially in velocity ranges close to the lower limit. In Figure 11b, measurements made with the red LED show a regression line with an R^2^ = 0.5696, explaining 56.96% of the variability in the data; it shows less data dispersion, particularly at higher velocity ranges; however, there is much dispersion at low velocities. In Figure 11c, measurements made with the yellow LED show a regression line with an R^2^ = 0.4238, explaining 42.38% of the variability in the data; however, there is also significant dispersion in the data both above and below the regression line, which might result in reduced error (they compensate) despite the large dispersion they exhibit. In Figure 11d, measurements made with the green LED show a regression line with an R^2^ = 0.5906, explaining 59.06% of the variability in the data; it has high accuracy at high velocities; however, there is significant data dispersion, especially at low velocities, which may be due to predicting velocity close to the system’s minimum operating limit, as well as the potential impact of the system’s accessories on the readings. Measurements made with the blue LED are shown in Figure 11e; there is less dispersion in the data similar to the white LED, compared to other colors, especially at both high and low-velocity ranges; the regression line has an R^2^ = 0.6484, which explains 64.84% of the variability in the data; this indicates that at these wavelengths, the accuracy of the system’s predicted velocity detection improves and reduces deviations at both low and high velocities.

**Figure 12:**
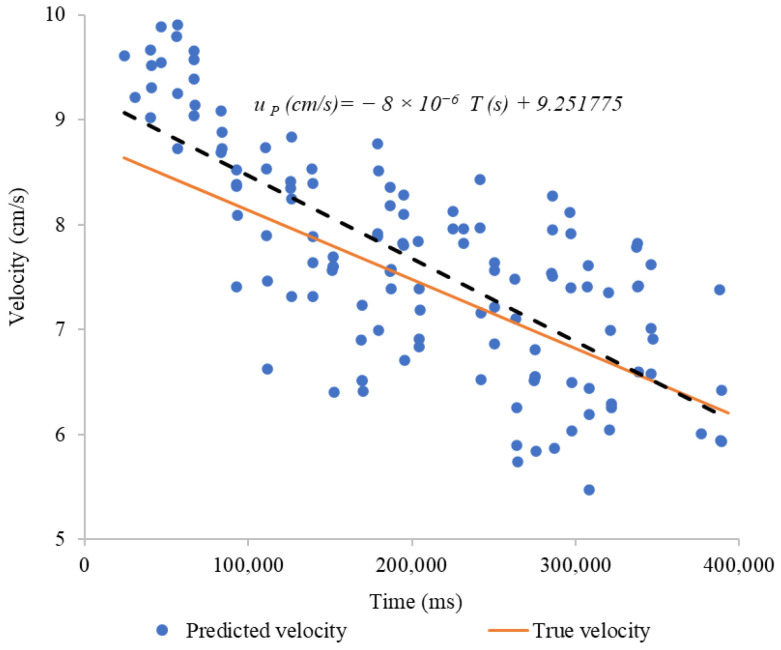
Predicted velocity vs. true velocity, potable water.

Figure 12 shows the predicted velocity vs. time. These data illustrate the trend observed in the measurements after processing the data with the proposed Algorithms A3–A5. Two important lines are highlighted: a dashed line representing the trend of the linear regression fit of predicted velocity with respect to time, which explains 54.50% of the variability of the data (R^2^ = 0.545), and an orange line representing the true velocity of the system.

In Table 3, it is shown that the readings with the lowest mean absolute and relative error were those from the blue LED in potable water, with a Mean Absolute Error (MAE) of 0.56 and a Mean Relative Error (MRE) of 7.59; in contrast, the readings from the green LED exhibited higher errors, with an MAE of 0.71 and an MRE of 9.58.

### 5.4. Calibration Results with Water with Chlorophyll

As previously mentioned, we used the amount of data vary in each color series, possibly due to compilation errors or some LDRs being more sensitive than others. Additionally, the color in which specific turbidity is better detected should be considered, as this may open up new avenues for research.

**Figure 13:**
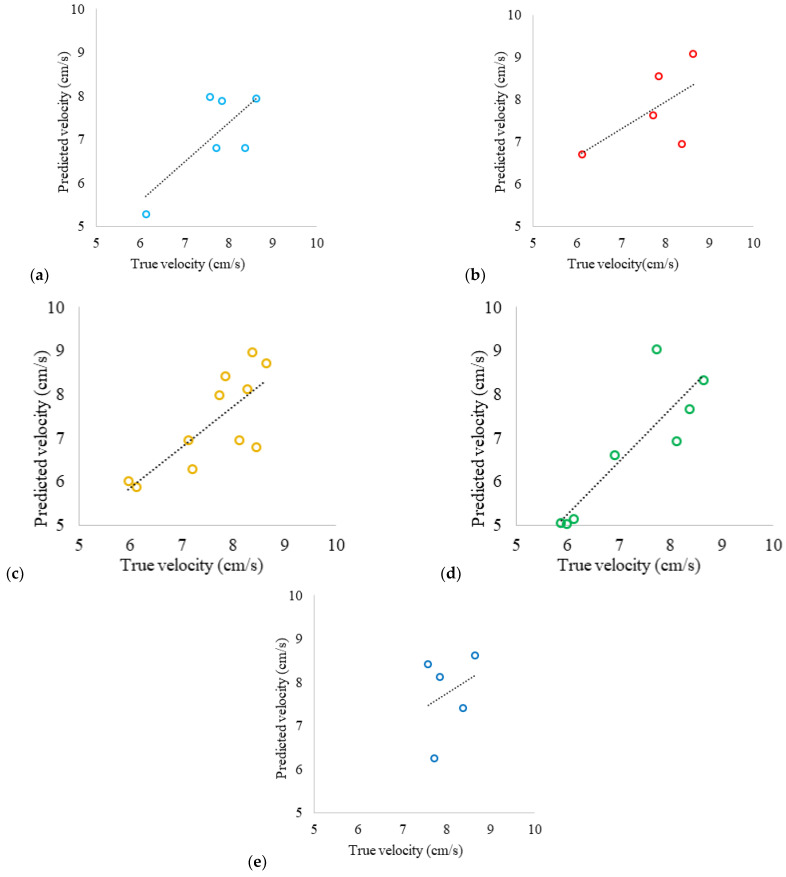
Predicted velocity vs. true velocity, water with chlorophyll: (**a**) white LED; (**b**) red LED; (**c**) yellow LED; (**d**) green LED; (**e**) blue LED.

In Figure 13, the results of predicted velocity (results of Algorithms A3–A5) versus true velocity for each of the colors used in water with chlorophyll are shown. The results reveal a positive slope with significant data dispersion across all series. However, the calculated velocities from the readings using the Yellow LED generally have fewer errors on average; as shown in Figure 13c, despite having greater dispersion, on average, it has less error within this wavelength range of the visible spectrum; the regression line has an R^2^ of 0.5883, which explains 58.83% of the variability in the data; it shows significant dispersion at high velocities; whereas at low velocities it has good correlation, with a slope of 0.93. In Figure 13a, it is observed that the calculations made with the data from the White LED mostly cluster close to the regression line; the regression line has an R^2^ of 0.5587, which explains 55.87% of the variability in the data; it shows dispersion in the data, especially at higher velocity ranges. Figure 13b shows that the calculations made with the data from the Red LED approach the regression line, with significant dispersion at high velocities; the regression line has an R^2^ of 0.382, which explains 38.20% of the variability in the data. In Figure 13d, it is observed that the calculations made with the data from the Green LED mostly cluster close to the regression line; the regression line has an R^2^ of 0.7631, which explains 76.31% of the variability in the data; it generally shows less dispersion compared to other colors; however, the slope is 1.2018, which deviates from the ideal trend compared to the Yellow LED. Figure 13e shows that the calculations made with the data from the Blue LED show significant dispersion; the regression line has an R^2^ of 0.0887, which explains 8.87% of the variability in the data.

**Figure 14:**
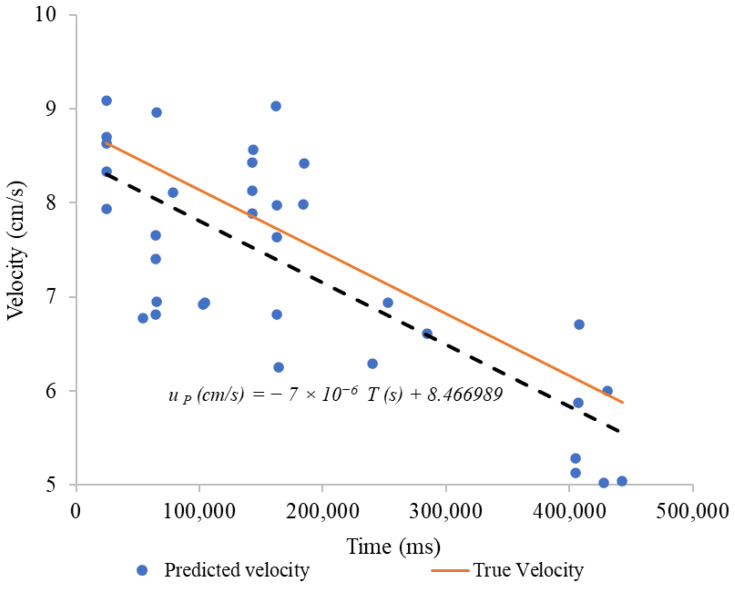
Predicted velocity vs. true velocity, water with chlorophyll.

Figure 14 shows that the regression line for predicted velocity has a notable similarity in both slope and intercept with the true velocity line. Although the predicted velocity points exhibit some dispersion, explaining 58.29% of the data variability (R^2^ = 0.5829). The linear regression has a slope of −6.576 × 10^−6^, in contrast to the slope of the true velocity, which is −6.6 × 10^−6^. The proximity between the two lines suggests that, on average, the proposed algorithm adequately predicts the true velocity.

In Table 4, it is shown that the readings with the lowest mean absolute error and mean relative error were those from the yellow LED in chlorophyll water, with an MAE of 0.54 and an MRE of 6.80. Conversely, readings from the green LED exhibited higher errors, with an MAE of 0.83 and an MRE of 11.83.

### 5.5. Model Testing in Simulated Runoff Fluid

The peaks in the figures (results of Algorithms A1 and A2) that will be shown next represent the time ranges during which the soil substrate dilutes into the water. The water does not immediately mix with the substrate, as there are moments when the mixing is low, maintaining reduced turbidity. However, at certain moments, a larger volume of substrate detaches from the soil and mixes in, increasing the turbidity. This latter mixing acts as a natural tracer, creating pronounced curves. The turbidity of the mixture was 60 NTU. These new readings exhibit characteristics similar to the measurements taken during calibration, demonstrating the expected temporal offset.

**Figure 15:**
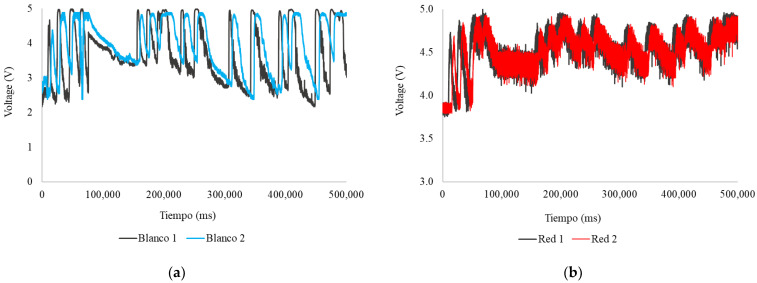
Readings during the sampling period, runoff: (**a**) white LED; (**b**) red LED.

Figure 15a shows the voltages resulting from changes in the absorption and scattering of light from the LED White during the sampling period (500,000 ms). The fluctuations in the readings generate 17 pronounced curves in both heads; the first head (White 1) has a maximum of 5.00 V, a minimum of 2.17 V, and an average of 3.64 V, while the second head (White 2) has a maximum of 4.89 V, a minimum of 2.38 V, and an average of 3.95 V. Figure 15b shows the voltages resulting from changes in the absorption and scattering of light from the LED Red during sampling. The fluctuations in the readings generate 17 pronounced curves; the first head (Red 1) has a maximum of 5.00 V, a minimum of 3.76 V, and an average of 4.55 V, while the second head (Red 2) has a maximum of 4.94 V, a minimum of 3.79 V, and an average of 4.53 V.

It is important to note that the readings presented in Figure 15b exhibited a significant noise level, like the LED Yellow. Despite this, it is observed that the readings maintain general consistency with the results from the other figures, although with poor resolution. This suggests that the readings might be affected by turbidity or particles, such as pieces of leaves or branches, which floated on the surface due to their lower density and obstructed readings. Other external factors that may have affected the readings should also be highlighted, such as data compilation errors or the influence of moisture, which may have compromised some of the sensor connections.

#### 5.5.1. Extended Runoff Reading

**Figure 16:**
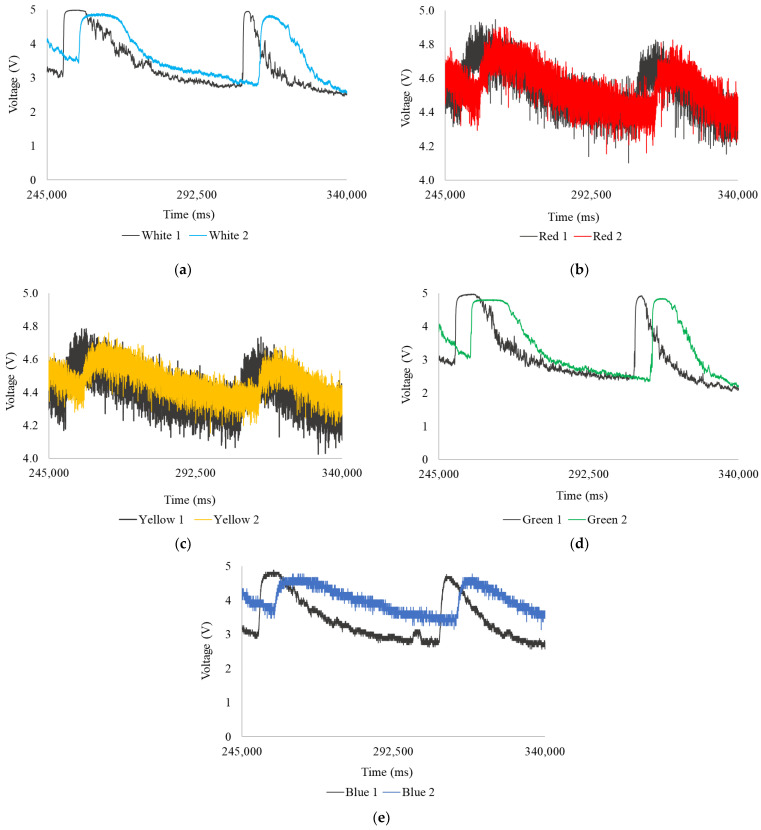
Extended readings from 245,000 ms to 340,000 ms, runoff: (**a**) white LED; (**b**) red LED; (**c**) yellow LED; (**d**) green LED; and (**e**) blue LED.

In Figure 16, the voltages resulting from Algorithms A1 and A2 show changes in light absorption and scattering between 245,000 ms and 340,000 ms. The measurements highlight the time lag between the curves with good resolution; the fluctuations tend to increase, generating two very pronounced curves.

In Figure 16a, in the readings from the first head (White 1), the first curve has a maximum of 4.99 V, a minimum of 3.03 V, and an average of 4.13 V; while the second has a maximum of 4.96 V, a minimum of 2.74 V, and an average of 3.76 V. In the second head (White 2), the first curve has a maximum of 4.89 V, a minimum of 3.26 V, and an average of 4.23 V; while the second has a maximum of 4.85 V, a minimum of 2.82 V, and an average of 4.35 V. In Figure 16b, in the readings from the first head (Red 1), the first curve has a maximum of 4.95 V, a minimum of 4.31 V, and an average of 4.66 V; while the second has a maximum of 4.82 V, a minimum of 4.24 V, and an average of 4.61 V. In the second head (Red 2), the first curve has a maximum of 4.90 V, a minimum of 4.30 V, and an average of 4.65 V; while the second has a maximum of 4.82 V, a minimum of 4.21 V, and an average of 4.60 V. In Figure 16c, in the readings from the first head (Yellow 1), the first curve has a maximum of 4.78 V, a minimum of 4.18 V, and an average of 4.51 V; while the second has a maximum of 4.73 V, a minimum of 4.11 V, and an average of 4.48 V. In the second head (Yellow 2), the first curve has a maximum of 4.76 V, a minimum of 4.29 V, and an average of 4.55 V; while the second has a maximum of 4.68 V, a minimum of 4.21 V, and an average of 4.51 V. In Figure 16d, in the readings from the first head (Green 1), the first curve has a maximum of 4.98 V, a minimum of 2.65 V, and an average of 3.80 V; while the second has a maximum of 4.93 V, a minimum of 2.42 V, and an average of 3.63 V. In the second head (Green 2), the first curve has a maximum of 4.80 V, a minimum of 2.83 V, and an average of 4.01 V; while the second has a maximum of 4.84 V, a minimum of 2.36 V, and an average of 4.19 V. In Figure 16e, in the readings from the first head (Blue 1), the first curve has a maximum of 4.89 V, a minimum of 2.95 V, and an average of 3.93 V; while the second has a maximum of 4.77 V, a minimum of 2.70 V, and an average of 4.03 V. In the second head (Blue 2), the first curve has a maximum of 4.78 V, a minimum of 3.48 V, and an average of 4.33 V; while the second has a maximum of 4.78 V, a minimum of 3.26 V, and an average of 4.33 V; additionally, the readings from the second head lose resolution due to the presence of particles, leaves, or branches that, having less density, rub against the inner surface of the pipe, obstructing the readings.

#### 5.5.2. Test Results

**Figure 17:**
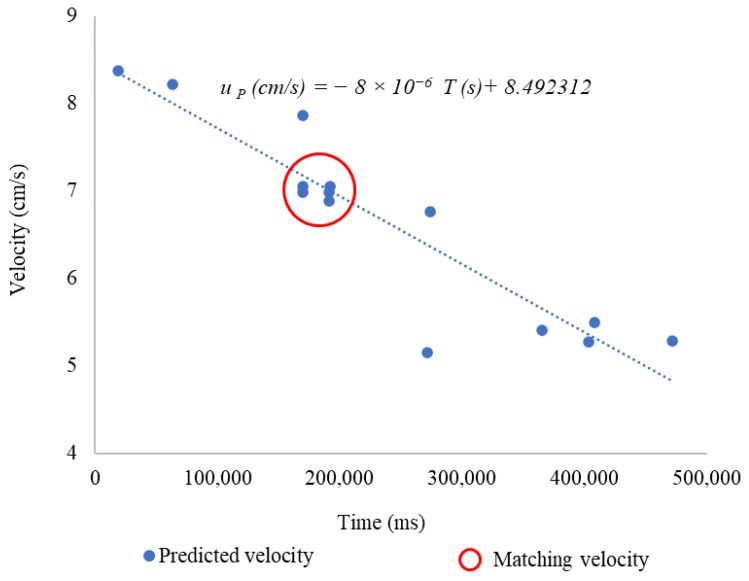
Predicted velocity vs. true velocity, runoff.

In order to obtain the results, we apply Algorithms A3–A5 (see Appendix A), considering velocity values derived from readings of the white, green, and blue LEDs. Red and yellow were not considered due to the significant amount of uncertainty they introduce. These results are shown in Figure 17. The measurements have significant consistency, especially in the interval from 160,000 ms to 200,000 ms, indicating that the sensor can determine the flow velocity with great precision. The regression line would explain the velocity behavior during sampling and 84.3% of the variability in the predicted velocity.

The matching of points is shown in Table 5, highlighting the accuracy and sensitivity of the sensor.

## 6. Discussion

**Figure 18:**
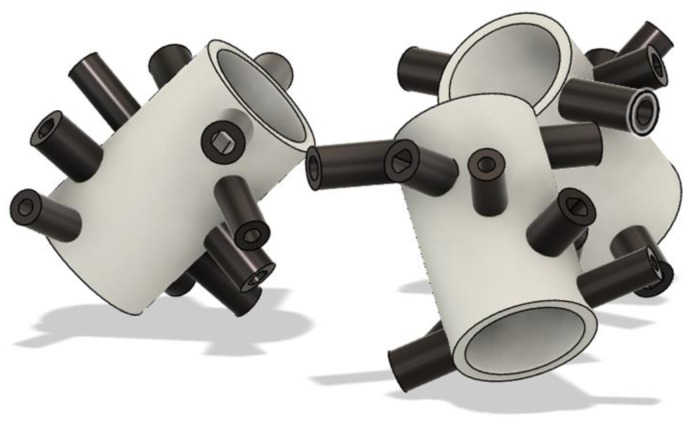
Distribution of sensors for three-dimensional velocity calculation.

The most well-known current meters are Acoustic Doppler Current Profilers, which can measure velocities at great depths and whose cost from different brands can easily exceed EUR 40,000. A device with similar characteristics to the sensor proposed in this work is the Vector 6 MHz from Nortek, priced at approximately EUR 22,000. The main similarity between these devices is that both require a monitoring station for measurements. Additionally, the result is a velocity vector indicating both magnitude and three-dimensional direction. This can be replicated in the proposed sensor by using three meters oriented at 45° elevation on the Z-axis and distributed 120° apart in the XY plane, as shown in Figure 18.

Comparing the proposed sensor with existing proposals for water-related parameters monitored with low-cost sensors, we can affirm that the performance of our sensors is aligned with the literature. Following, we summarize some of the most recent proposals and their performance. In [33], the authors monitored the water level using an Arduino and achieved accuracy, which was characterized by an R2 equal to 0.5 to 0.9 for the different experiments. Several cases are proposed for turbidity monitoring, which is characterized by R2 values higher than 0.9 [34,35]. Other authors have compared the performance of commercial low-cost sensors with professional devices, attaining R2 around 0.8 [36]. The use of ISFET sensors for pH monitoring has been proposed with unclear results [37]. Meanwhile, other authors [38] used a virtual for the same purpose; nevertheless, its performance was not evaluated with R2 since the sensors were used to classify data instead of performing a regression.

The following examples were found focusing on the use of sensors for water flow or velocity. Some authors have proposed optical fiber sensors for water velocity measurement [39], while others have proposed the use of hall effect sensors [23] or pressure sensors [40]. None of them indicated the R2 of their proposals, which makes it impossible to compare. Another option was presented in [41] based on the use of thermocouples and a heater [42]. The use of water flow sensors based on gamma radiation has been proposed in [42], achieving an R2 of 0.9. Recently, a virtual sensor has been proposed for the same purpose, combining other physical sensors with machine learning [43], and the obtained R2 attained values higher than 0.9. The proposed sensor’s maximum R2 was 0.76 in controlled conditions and 0.84 in real scenarios. It must be noted that even though this value is slightly inferior to the current proposals, the system was tested in real conditions, and it is characterized by a low-cost compared with the other proposals.

Table 6 presents the cost of the sensor components, which is appealing. This velocity sensor will be one of the components included in [44] for continuous monitoring. It is important to clarify that the sensor will not be directly submerged in water; it will have appropriate encapsulation. The sensor housing will have a fusiform shape to minimize its impact on the water flow.

In the next prototypes and applications, the use of an RGB LED that allows the selection of the best light will be considered. Thus, according to a preliminary analysis of the scenario, the system will automatically select the color with better performance for the encountered conditions, such as using blue LED in freshwater or yellow LED in chlorophyll-containing water. This way, since fewer lights are used, the sensor will become adaptable, reduce the required time for sampling, and enhance the energy efficiency of the devices. Moreover, it is possible that by diminishing the time interval between light measurements, it will become easier to measure higher water flow velocities.

## 7. Conclusions

This work proposes a low-cost optical sensor for measuring flow velocities in aquatic environments based on LEDs and LDRs to detect variations in light dispersion and absorption in water. The structural design and processing algorithm have proven to be efficient, providing accurate readings under various turbidity conditions and predicted velocity. It is worth noting that the sensor can operate at relatively high turbidity levels compared to those found in coastal waters due to water turbulence. This methodology minimizes direct interaction between the sensor and the fluid being measured, contributing to minimal maintenance and long-term monitoring. The proposed sensor, in addition to being effective, is extremely economical compared to commercial alternatives, representing less than 0.43% of the cost of a Nortek Vector 6 MHz and 0.21% of the Teledyne Workhorse II 300 kHz Marine, making it a valuable tool for sustainable marine ecosystem management.

Future research will focus on conducting various tests with different distributions of visible light wavelengths to prevent interference between them, as well as developing a more compact single sensor to reduce the measurement distance and avoid the diffusion of natural tracers present in the sampling medium. Another important aspect to consider is the influence of ambient light on sensor readings. An innovative aspect is the potential use of the spectral signature obtained by the sensor to identify and distinguish the presence of contaminants or specific water components, such as chlorophyll concentration, sediments, or other organic compounds, as performed in [45]. An RGB LED will be implemented to cover the full-color spectrum, allowing for the analysis of velocity and dispersion phenomena under various conditions. This approach will not only enable the measurement of flow velocity but also allow for the study of water quality and composition. It would provide a valuable tool for monitoring aquatic ecosystems.

## Figures and Tables

**Table 1:** Table of system velocity reading.

No.	Measurement Time (s)	Measured Volume (cm^3^)	Flow Rate (cm^3^/s)	Velocity (cm/s)	Time (s)
1	21.06	930	44.15	8.71	13.06
2	23.24	990	42.61	8.41	59.26
3	23.68	925	39.06	7.71	165.26
4	24.08	905	37.58	7.42	209.45
5	26.11	942	36.08	7.12	254.53
6	28.90	992	34.32	6.77	307.07
7	29.11	948	32.57	6.43	359.53
8	30.20	920	30.46	6.01	422.38
9	33.87	962	28.40	5.61	483.97

**Table 2:** Experimental fluids.

Fluid	Turbidity (NTU)
Potable water	2
Water with chlorophyll	10
Runoff	60
Tracer	25

**Table 3:** Mean absolute and relative errors with different LEDs and potable water.

LEDs	Mean Absolute Error (cm/s)	Mean Relative Error (%)
White	0.70	9.23
Red	0.62	8.36
Yellow	0.58	8.04
Green	0.71	9.58
Blue	0.56	7.59
Average	0.63	8.56

**Table 4:** Mean absolute and relative errors in different LEDs, water with chlorophyll.

LEDs	Mean Absolute Error (cm/s)	Mean Relative Error (%)
White	0.74	9.68
Red	0.65	8.44
Yellow	0.54	6.80
Green	0.83	11.83
Blue	0.71	9.02
Average	0.69	9.15

**Table 5:** Matching predicted velocity data, runoff.

Time (ms)	Predicted Velocity (cm/s)	LED
169,705	7.05	Blue
170,105	6.99	White
190,855	6.89	Blue
190,909	6.99	Green
191,727	7.05	White

**Table 6:** Cost of the proposed sensor.

Components	Cost (EUR)
Arduino Mega 2560 Rev3	51.20
LEDs	5.00
LDRs	5.00
Resistors	4.00
Cables	5.00
Breadboard	10.00
Transparent pipe	5.00
Housing	10.00
Total	95.20

## Data Availability

The data provided can be found within the article. The original contributions made in this study are included in the document; any additional inquiries can be directed to the author or authors responsible.

## Appendix A

**Algorithm A1: Sampling (raw data)**
const int LED [10] = {4, 5, 6, 7, 8, 9, 10, 11, 12, 13}; const int LDR [10] = {A0, A1, A2, A3, A4, A5, A6, A7, A8, A9}; unsigned long ciclo = 0; void setup() { for (int i = 0; i < 10; i++) { pinMode(LED[i], OUTPUT); digitalWrite(LED[i], HIGH); pinMode(LDR[i], INPUT);} Serial.begin(574880); } void loop() { ciclo++; unsigned long tiempoActual = micros(); Serial.print(ciclo); Serial.print(“, “); Serial.print(tiempoActual); for (int i = 0; i < 10; i++) { Serial.print(“, “); Serial.print(analogRead(LDR[i]));} Serial.println(); delay(6.4); }

**Algorithm A2: Voltage conversion**
data=Water; error_margen = 0.2; CI= data(:, 2:11); new_max = 5; max_col = 1023; normalize_volt = @(col, max_col, new_max) ((col / (max_col)) * new_max; normalized_columns = arrayfun(@(col_idx) normalize_volt(CI(:, col_idx), max_col, new_max), 1:size(CI, 2), ‘UniformOutput’, false); normalized_columns = cell2mat(normalized_columns); data(:, 2:11) = normalized_columns;

**Algorithm A3: Removal of liner trend**
CI = arrayfun(@(col_idx) detrend(CI(:, col_idx), “constant”), 1:size(CI, 2), ‘UniformOutput’, false); CI = cell2mat(CI); CI = arrayfun(@(col_idx) max(CI(:, col_idx), 0), 1:size(CI, 2), ‘UniformOutput’, false); CI = cell2mat(CI); data(:, 2:11) = CI;

**Algorithm A4: Local máxima determination**
x1 = data(:, 1); LM = double(islocalmax(CI, 3000, LocalMax)); sums = LM(:, 1:5) + LM(:, 6:10); TF = arrayfun(@(i) [x1, CI(:, i), CI(:, i + 5)], 1:5, ‘UniformOutput’, false); TF = cellfun(@(tf, sum) tf(sum > 0, :), TF, num2cell(sums, 1), ‘UniformOutput’, false); [TF1, TF2, TF3, TF4, TF5] = TF{:};

**Algorithm A5: Speed calculation**
function new_data = create_new_matrix(TF) TF = [zeros(1, size(TF, 2)); TF]; num_rows = size(TF, 1); num_cols = size(TF, 2); new_cols = zeros(num_rows, 7); for i = 2:num_rows-1 if num_cols >= 2 && TF(i, 2) ~= 0 && TF(i + 1, 2) == 0 new_cols(i, 1) = 1; end if num_cols >= 3 && TF(i, 3) ~= 0 && TF(i + 1, 3) == 0 new_cols(i, 2) = 1; end end for i = 2:num_rows if new_cols(i, 1) == 1 new_cols(i, 3) = TF(i, 1); end if new_cols(i, 2) == 1 new_cols(i, 4) = TF(i, 1); end end for i = 2:num_rows if new_cols(i-1, 4) > 1 new_cols(i, 5) = 0; elseif new_cols(i-1, 5) == 0 if new_cols(i-1, 3) == 0 new_cols(i, 5) = 0; else new_cols(i, 5) = new_cols(i-1, 3); end else new_cols(i, 5) = new_cols(i-1, 5) + 1; end end for i = 1:num_rows if new_cols(i, 4) > 1 && new_cols(i, 5) > 1 new_cols(i, 6) = abs(new_cols(i, 4) − new_cols(i, 5)); else new_cols(i, 6) = 0; end end for i = 1:num_rows if new_cols(i, 6) ~= 0 new_cols(i, 7) = 46.5 / new_cols(i, 6) * 1000; else new_cols(i, 7) = 0; end end new_data = [TF, new_cols]; end
